# Cholinesterase Research Outreach Project (CROP): measuring cholinesterase activity and pesticide use in an agricultural community

**DOI:** 10.1186/s12889-015-2076-8

**Published:** 2015-08-05

**Authors:** Jacqueline Cotton, Paul Lewandowski, Susan Brumby

**Affiliations:** National Centre for Farmer Health, Western District Health Service, PO Box 823, Hamilton, Vic 3300 Australia; School of Medicine, Deakin University, 75 Pigdons Road, Waurn Ponds, Vic 3216 Australia

**Keywords:** Farmer, Organophosphate, Pesticides, Safety, Chemical, Acetylcholinesterase

## Abstract

**Background:**

Australian farmers and their workers are exposed to a wide variety of pesticides. Organophosphate (OP) insecticides are a widely used class of pesticide used for animal husbandry practices (Naphthalophos for sheep dipping, jetting and drench), crop production for pest control (Dimethoate) and in public health (Maldison for head lice). Acute poisonings with this class of insecticide are reported among agricultural workers and children around the globe, due to the inhibition of acetylcholinesterase (AChE). Less is known about chronic exposures. Regular monitoring of erythrocyte AChE will enable farmers to identify potential exposure to organophosphate insecticides and take action to reduce exposures and improve their health and safety practices. This study aims to assess and improve the integration of AChE monitoring into routine point of care health clinics, and provide farming and non-farming people with a link between their AChE activity and their household chemical and agrichemical use.

**Methods/Design:**

The research will target individuals who work on mixed farming enterprises and routinely using OPs (*n* = 50) and non-farmers (*n* = 30). Baseline data are collected regarding demographic, health conditions and behaviours, Kessler 10 (K10) scores, chemical use and personal protection. Baseline anthropometric measures include height, weight, hip and waist circumference, body fat analysis and, biochemical analysis of fasted total serum cholesterol, triglycerides, low-density cholesterol (LDL), high-density cholesterol (HDL) and blood glucose. Analysis of erythrocyte cholinesterase (EAChE) activity is also conducted using a finger prick test. Testing of EAChE is then repeated in all participants every 3 weeks for a maximum of three times over a period 10 weeks. Participants are provided with full feedback and counselling about their EAChE activity after each reading and a detailed summary provided to all participants at the completion of the study. Data will be analysed using repeated measures within a general linear model.

**Discussion:**

This work will provide an evidence base and recommendations for the integration of EAChE monitoring into Australian rural health clinics, leading to research which will further quantify pesticide exposure both on the farm and in the home, highlighting the importance of sustaining and providing a safe work and home environment for farming communities.

**Trial registration:**

ACTRN12613001256763

## Background

Australian farmers and their workers are exposed to a wide variety of pesticides [1]. Pesticides are substances that destroy, repel or attack pests that have a negative effect on productivity and profitability of a farming enterprise. Pesticide groups include herbicides, insecticides, fungicides and rodenticides. Organophosphate (OP) insecticides are widely used for animal husbandry practices (Naphthalophos sheep drench), public health (head lice, locust control), and in crop production (Dimethoate for insect control). The OP class of insecticides effect the nervous system with poisonings most common among agricultural workers and children [2, 3]. OPs are regularly used as a form of pest control on most farms and farmers have enhanced their understanding of pesticide use and handling. However, many are still not meeting the full range of personal protection standards in their own handling and application [4]. Routes of human exposure include dermal absorption, inhalation and ingestion [5]. Symptoms of OP toxicity are caused by inhibition of the enzyme acetylcholinesterase (AChE). Inhibition of AChE results in the subsequent accumulation of acetylcholine at the cholinergic synapses of nerves causing uncontrolled firing of the synapse [1, 6]. Subclinical effects of chronic OP exposure such as AChE inhibition may be detected early by biological tests. Recovery from AChE depression is prolonged, due to the irreversible binding of OPs to the red blood cell and its subsequent 120 day life cycle [7]. Monitoring exposure to organophosphates involves the measurement of peripheral cholinesterase enzymes that are inhibited by organophosphates. These include; erythrocyte cholinesterase (EAChE) and serum cholinesterase (SAChE). Organophosphates have been associated with chronic neurological disease such as impaired memory, impaired fine motor skills control and Parkinson’s disease [8]. The evidence suggests that whilst an acute episode experience may not have occurred, exposure over a long period of time (such as in workers dipping sheep) may result in neurological conditions such as Parkinson’s disease and neuropsychiatric conditions [6, 8, 9]. The window for exposure to toxicants may occur years before the onset of neurological symptoms [8, 10, 11], this is particularly important for sheep and crop producers exposed to long-term, low levels of OPs. The Western region of the state of Victoria in Australia has been a centre of sheep production, since settlement, and a large number of farmers have been exposed to OPs through routine sheep dipping (lice control), drenching (control internal parasites), jetting (blow fly control) and spraying of pastures (insect control). The National Centre for Farmer Health (NCFH) is in a unique position to monitor these farmers and workers, and work with farmers to incorporate cholinesterase testing as part of their assessments during peak periods and provide much needed data for them on exposures in this area. Additional practices such as mixing contaminated laundry and storing pesticides in the home are not only common causes of exposure to the farmer and farm worker, but may also be placing other household members at risk, particularly children [12]. Work completed by the program Sustainable Farm Families™ [13] has identified that there is strong interest from farm men and women who wish to investigate exposures and possible health impacts of agricultural chemical use.

The acute effect of OP exposure is well documented in Australia and overseas for humans, pests and animals [3, 6, 9]. However, the extent to which asymptomatic monitoring is taking place is not well known or documented. This research builds on work conducted by the NCFH. It utilises the data collection methods of the Sustainable Farm Families™ which includes health behaviours and conditions, with the expectation that this monitoring EAChE testing will be integrated into this procedure, providing the springboard to break through barriers that are currently inhibiting best practice chemical handling safety in Australia.

Understanding the mode of action of a chemical is a critical component of the risk assessment process. Cholinesterase activity is a direct link to exposure and this pilot study will produce valuable data on possible exposures for farmers routinely using OP’s for animal husbandry or crop production.

Monitoring cholinesterase levels during agricultural health screening and assessment is a key objective of Agrisafe™ clinics in Australia. However, its incorporation into such clinics will depend on health professional’s skills and knowledge. This study will further prepare health care providers for integration of correct cholinesterase testing procedures into point of care (POC) health checks. This work will further highlight pesticide exposure as a risk for farmers, workers and their families, leading to an easily adopted method that will quantify the environmental exposures providing early detection. Research has also shown links between cholinesterase activity and vascular complications in diabetic patients [14]. Diabetes is a disease also of increasing concern to the farming and rural population in Australia [15].

### Aims

The Cholinesterase Research Outreach Project (CROP) will use clinical sampling to determine the level of exposure associated with the routine use of OPs by Victorian farmers and/or their workers. This preliminary study will examine the methodology of testing for EAChE activity at POC and provide evidence for future work by identifying the exposure patterns and pathways of individuals most at risk in farming communities. It is anticipated that CROP will inform future research studying pesticide exposure in farming communities.

## Methods/design

### Evaluation design

To evaluate if a difference in cholinesterase activity was present in farmers before and after exposure, sample size is determined at the power of 80 % (β = 0.2) with α set at 0.05 for an effect size of one standard deviation. This requires the sample size to be a minimum of 30 farming individuals exposed to pesticides. By considering the probable retention rate (≥85 %) for a 7-month study, based on previous research in similar populations, [16] the target recruitment number is fixed at a target of 50 farmers.

### Sample size and inclusions

Consent will be obtained from 80 participants recruited via existing contacts and industry groups, letterbox drops and newspaper articles from a number of farming and community groups, specifically;

Mixed farming enterprises and sheep dipping/spraying contractors (*n* = 50) who are using OPs and a convenience group of non-farming individuals (*n* = 30) from the rural community of Hamilton, Australia.

Participants need to have been farming for more than 5 years, be aged between 18 and 75 years, speak English, and not had a previous known chemical incident. Questionnaires will be used to determine whether or not they are the primary pesticide user. Any non-farming individuals tested must also be aged between 18 and 75 and have not had a previous chemical incident. Participants from both groups will be self-reported not pregnant, not suffering from a known chronic disorder, not taking anti-inflammatory drugs or supplements and not exercising excessively throughout the study.

### Anthropometry

The simplicity of anthropometry allows it to be used in population-based studies to assess body-composition changes over time, as well as in clinical and field situations where access to technology is limited such as in this study. Height, weight, waist and hip circumference has previously been included as part of an initial POC assessment for farm men and women and agricultural workers. Participants have found these measurements helpful and easy to understand and also cite having a free health assessment as a reason to attend [13]. Additionally, (whilst not the focus of this study) if used in combination with blood biochemistry and body fat percentage, anthropometry can identify distinct fat distribution changes that occur in the elderly [17]. Weight is measured to the nearest 0.05 kg using electronic scales taken in light clothing with shoes removed and pockets emptied and prior to breakfast. Height is measured using a portable stadiometer to the nearest 0.1 cm with shoes removed and weight distribute evenly on both feet.

### Physical assessments and health condition data

**Fig. 1:**
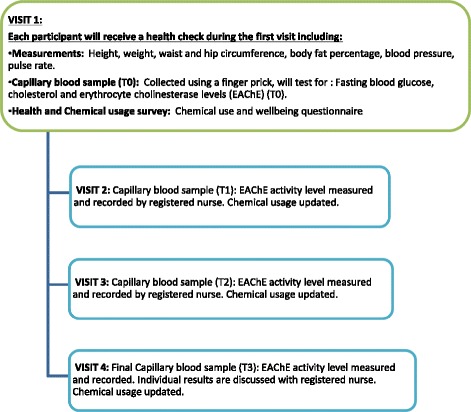
Point of care schedule for cholinesterase assessment

A short health history and health behaviours (smoking and alcohol consumption habits) will be obtained from participants with baseline physical assessments of blood pressure measurement (repeated twice), pulse rate, body fat percentage (bioelectrical impedance), respiratory function test (PiKo meter) also measured (see Table 1). Biochemical analysis of EAChE activity using a 10 μl capillary blood sample (measured by Test-mate ChE Cholinesterase tester system Model 400), and EAChE field Assay kit - to establish a baseline reading, will be used (Fig. 1). The Test-mate field assay testing system is based on the Ellman method [18]. Blood glucose and lipids (LDL, HDL) and total cholesterol is also taken after 10-hour fast using the same blood sample.

**Table 1:** Data collection description and timeline for farmer and non-farmer groups

Variable	Baseline (T0)	Visit 1 (T1, 1 mth)	Visit 2 (T2, 2 mth)	Visit 3 (T3, 3 mth)
Body weight	✓	X	X	X
Height	✓	X	X	X
Waist circumference	✓	X	X	X
Hip circumference	✓	X	X	X
Body fat %	✓	X	X	X
Blood pressure	✓	X	X	X
Pulse rate	✓	X	X	X
Fasting blood glucose	✓	X	X	X
Fasting blood cholesterol	✓	X	X	X
Fasting triglycerides	✓	X	X	X
HDL cholesterol	✓	X	X	X
LDL cholesterol	✓	X	X	X
Erythrocyte Cholinesterase (EAChE)	✓	✓	✓	✓
Description of monthly chemical use	✓	✓	✓	✓
Kessler (K10)	✓	X	X	X
Chemical Usage Survey	✓	X	X	X

Waist circumference is measured to the nearest 0.1 cm at the end of a normal expiration using a constant tension “Figure Finder Tape Measure”™ [19]. Body Mass Index will be calculated to classify obesity and overweight cut off points for future reference using the WHO parameters [20].

### Survey methodology

Trained research staff will collect all anthropometric and behavioural data. Sociodemographic data are collected directly from the participant including age, gender, country of origin, using the Victorian Department of Health service coordination tools (SCOT) [21]. The type of farming undertaken and residential postcode will also be collected from the participants.

A validated questionnaire, the Kessler 10 (K10) will be used to measure psychological distress. The K10 is a 10-item questionnaire intended to yield a global measure of distress based on questions about anxiety and depressive symptoms that a person has experienced in the most recent 4 week period [22]. This survey has been used extensively in Australia and provides a comparison to the general population. Given the relationship between organophosphates, neuropsychological and psychiatric functioning a baseline was deemed important [23, 24]. It also forms part of the usual farmer health assessment.

### Occurrence of illness & injury

Occurrence of illness or injury experienced within the last 3 months will be measured using a questionnaire describing 33 symptoms of chemical exposure, in random order of severity. The illness and injury data will be standardised and analysed accordingly.

### Monitoring agrichemical use

**Fig. 2:**
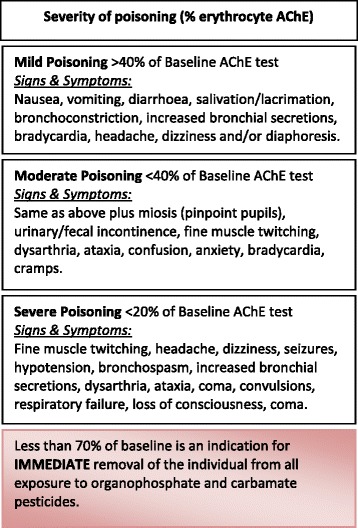
Guide to severity of acute poisoning with anti-cholinesterase agents (Source: Modified from California Environmental Protection Agency 2002)

In addition to completion of the agrichemical use survey of 13 questions, self-reported chemical use is recorded during each visit. Participants are provided with counselling regarding their EAChE levels in accordance with AgriSafe guidelines (Fig. 2). The USA’s Department of Pesticide Regulation specifies that a drop to 70 % or lower in red blood cell AChE or to 60 % or lower in plasma cholinesterase relative to the individual’s baseline is an indication for immediate removal of the individual from all exposure to organophosphate and carbamate pesticides [25]. Participants will be provided with a summary of their EAChE baseline and subsequent results at the conclusion of the study. This methodology reflects recent exposures and is used to provide feedback at the time of testing. However, it is noted that the level of current exposure is not a measure of long-term exposure and that the clinical parameters around long-term exposure are not well defined.

### Data entry, handling and statistical analysis

All data will be managed and analysed within the statistical program SPSS (IBM Corp. Released 2012 IBM SPSS Statistics for Windows, Version 21.0. Armonk, NY). SPSS is a statistical package widely used in quantitative social science research and is suitable for multivariate analysis. Data will be analysed using repeated measures (subjects as own controls) within the General Linear Model. Correlation coefficients between pesticide exposure and metabolic variables will be calculated using Pearson’s and Spearman’s correlation as appropriate. P values will be considered statistically significant at *p* < 0.05.

### Consent and ethics

All adults participating in the study are to be provided with a plain language statement and will provide informed written consent. Ethics approval has been granted to the project by Deakin University Human Research Ethics Committee (HREC 2013-100 dated 18/06/2013). All researchers involved in data collection have a Victorian Police check undertaken.

## Discussion

Organophosphates are still used widely with the actions and attitudes of farmers and workers using these pesticides is vital to their wellbeing and that of their families. It is common for mild to chronic depression of EAChE activity to be reported as ‘normal’ due to the wide reference range for EAChE activity [26]. It is important that farmers and agricultural workers who are routinely using OPs establish their baseline EAChE activity and have access to regular EAChE activity checks for comparison with baseline. Routine monitoring of EAChE may allow for early recognition of frequent and continuous low-level exposure to OPs. This work will provide an evidence base, leading to research to further quantify pesticide exposure both on the farm and in the home of farming families and highlight the importance of sustaining and providing a safe work environment for farming communities.

## Abbreviations

AChE: Acetylcholinesterase
EAChE: Erythrocyte Acetylcholinesterase
CROP: Cholinesterase Research Outreach Project
OP: Organophosphate
K10: Kessler 10
LDL: Low Density Lipoproteins
HDL: High Density Lipoproteins
NCFH: National Centre for Farmer Health
POC: Point of Care
SCOT: Service Coordination Tools
BMI: Body Mass Index
mth: Month
